# Chronotype-Dependent Changes in Sleep Habits Associated with Dim Light Melatonin Onset in the Antarctic Summer

**DOI:** 10.3390/clockssleep1030029

**Published:** 2019-08-07

**Authors:** Ana Silva, Diego Simón, Bruno Pannunzio, Cecilia Casaravilla, Álvaro Díaz, Bettina Tassino

**Affiliations:** 1 Laboratorio de Neurociencias, Facultad de Ciencias, Universidad de la República, 11400 Montevideo, Uruguay; 2 Unidad Bases Neurales de la Conducta, Instituto de Investigaciones Biológicas Clemente Estable, 1600 Montevideo, Uruguay; 3 Área Inmunología, Departamento de Biociencias (Facultad de Química) and Cátedra de Inmunología, Instituto de Química Biológica (Facultad de Ciencias), Universidad de la República, 11600 Montevideo, Uruguay; 4 Sección Etología, Facultad de Ciencias, Universidad de la República, 11400 Montevideo, Uruguay

**Keywords:** Antarctica, DLMO, MEQ, circadian preferences

## Abstract

Dim light melatonin onset (DLMO) is the most reliable measure of human central circadian timing. Its modulation by light exposure and chronotype has been scarcely approached. We evaluated the impact of light changes on the interaction between melatonin, sleep, and chronotype in university students (*n* = 12) between the Antarctic summer (10 days) and the autumn equinox in Montevideo, Uruguay (10 days). Circadian preferences were tested by validated questionnaires. A Morningness–Eveningness Questionnaire average value (47 ± 8.01) was used to separate late and early participants. Daylight exposure (measured by actimetry) was significantly higher in Antarctica versus Montevideo in both sensitive time windows (the morning phase-advancing and the evening phase-delaying). Melatonin was measured in hourly saliva samples (18–24 h) collected in dim light conditions (<30 lx) during the last night of each study period. Early and late participants were exposed to similar amounts of light in both sites and time windows, but only early participants were significantly more exposed during the late evening in Antarctica. Late participants advanced their DLMO with no changes in sleep onset time in Antarctica, while early participants delayed their DLMO and sleep onset time. This different susceptibility to respond to light may be explained by a subtle difference in evening light exposure between chronotypes.

## 1. Introduction

The biological clock has evolved in all living beings to adapt their functioning to a predictable light–dark cycling environment. Consequently, although one of the most essential characteristics of the biological clock is to keep a circadian time reference even in free-running conditions, the daily adjustment of the clock is very sensitive to light exposure [1,2]. Undoubtedly, light is the most important zeitgeber and entrainer of the clock across evolution [3,4,5]. It is indisputable that modern urban life has affected human light exposure by increasing it during nighttime (due to electric light and screens) and decreasing it during daytime (due to indoor work in enclosed buildings). It is a matter of current debate how the human clock copes with these quite recent changes in environmental light, by which the reliable day–night natural cycle has been distorted [6,7,8]. 

Human life schedules are more frequently organized to respond to family, work, and social demands than to the environmental light cycle. The pineal secretion of melatonin acts as the main signal for human integrative time keeping as indicator of the beginning of the night [9,10]. Though melatonin always shows a nocturnal increase even in people that remain active at night (for example, in nocturnal-shift workers [11,12]), the secretion of melatonin is highly sensitive to light. Light exposure at night suppresses melatonin secretion [13], but bright light at daytime (especially in the morning) increases nocturnal melatonin secretion [14,15]. In addition, the rhythm of melatonin secretion provides the best available measure of the timing of the internal clock [9]. The dim light melatonin onset (DLMO, the start of the evening rise of melatonin in plasma or saliva) is the best proxy of the functioning of the circadian clock in humans [10,16]. The DLMO itself is affected by light, so it can be either advanced by bright light exposure in the morning [17,18,19,20] or delayed by light exposure during the evening [21,22]. Likewise, it is clear that seasonal changes in day length affect the duration and phase of the nocturnal melatonin pulse [23,24]. 

Circadian preferences represent individual differences among people in the sleep–wake schedule, which depend on genetic factors, sex, and age, as well as on physical and social environmental cues [25,26,27,28]. The adult population can be classified into three chronotypes: Morning-oriented individuals (early chronotypes) that go to bed and wake up early, evening-oriented individuals (late chronotypes) that tend to remain active at night, and intermediate chronotypes that correspond to about 60% of the population [29,30]. Circadian preferences impact on the daily melatonin profile. Early melatonin onset is associated with early chronotypes, while late chronotypes show later DLMO values [31,32,33]. In addition, light exposure has been reported to influence circadian preferences completing the picture of mutual interactions between light, melatonin, and circadian preferences. In Amazonian rubber-tapper workers, later chronotypes and delayed melatonin onset occur in individuals exposed to electric light with respect to those exposed to the natural photoperiod [34].

Two previous studies evaluated the influence of acute light manipulation on the phase relationship between melatonin onset, sleep schedule, and circadian preferences [35,36]. Wright et al. [35] compared subjects under two conditions (constructed environment with electrical lighting and summer natural light–dark cycle camping) and found that not only do sleep patterns and melatonin profiles change and synchronize to solar time but also that later chronotypes exhibit larger circadian advances when exposed to only natural light. On the other hand, Figueiro et al. [36] found that the circadian phase and sleep patterns change in response to controlled advancing and delaying light exposure patterns regardless of individual chronotype. In the present study, we add evidence on the impact of light changes on the triple interaction between melatonin, sleep, and chronotype using the extreme light exposure of the Antarctic summer as an advantageous scenario. We observe chronotype-dependent changes in the melatonin profile and sleep schedule of Uruguayan university students when comparing their Antarctic summer trip with their normal life situation around the autumn equinox in Montevideo, Uruguay. 

## 2. Study Population and Methods

### 2.1. Participants

Twelve healthy students (3 males, 9 females) from the Facultad de Ciencias, Universidad de la República, Uruguay, were selected to participate in the Second Uruguayan Summer School on Introduction to Antarctic Research, held in January 2016 at the Uruguayan Antarctic Scientific Base Artigas. The impact of light and chronotype preferences on their sleep habits and melatonin levels was compared between the start of university semester in the fall equinox 2016 (March 7–17, 2016, Montevideo, Uruguay, 34°54′ S; 56°11′ W, LD 12:12) and the 2016 Uruguayan Summer Antarctic School (January 17–27, 2016, King George Island, 62°11′ S; 58°52′ W, LD 20:4). All participants were clinically assessed to confirm they met the required health conditions. The mean age of the participants was 22.58 ± 1.16 years old; none showed sleep disturbances or signs of depression (Beck Depression Inventory score <10, [37]). All procedures were approved by the ethics committee at the Instituto de Investigaciones Biológicas Clemente Estable, Ministerio de Educación y Cultura, Uruguay (CEP/HCPA 14-0057; approval date: 16 December 2013). All participants signed an informed consent form stating that they had been told about the aims and procedures of the study, their right to end participation without any explanatory statement at any time, their data being coded so that data evaluation could be carried out on an anonymous basis, and their data being communicated for scientific purposes only.

We considered Montevideo data as the control situation (in the sense that Montevideo represented their ordinary lives) for all the purposes of this study. In Montevideo, the students were evaluated at the beginning of the university semester, which means a presumably similar academic challenge to the summer Antarctic school. However, in addition to attending university courses, they were engaged in diverse work and entertainment activities with no restrictions nor special instructions. However, while participants in Montevideo were living apart in their regular lives, in Antarctica, all the participants were living together and followed the same daily agenda (meal time, type of food intake, activities, etc.).

### 2.2. Questionnaires

Chronotypes were assessed using both the Spanish version of the Munich Chronotype Questionnaire (MCTQ) [38] and the Spanish adaptation of the Morningness–Eveningness Questionnaire (MEQ [39]). The questionnaires were answered individually by all the participants during the first day of the Montevideo sample (7 March 2016). Validated MCTQ reports were used to assess the mid-sleep point on free days corrected for sleep debt on work days (MSFsc) as a proxy of individual chronotype [40,41]. The MEQ score, calculated from the answers about preferred sleep time and daily performance inquired in the MEQ, was also considered as proxy of individual chronotype, with higher scores indicating greater morningness tendencies [39,42]. Given that MEQ scores and MSFsc correlated nicely, we only used the MEQ score as an indicator of participants’ circadian preference throughout. Based on the average value of their MEQ score, participants were categorized into two groups: Late chronotypes, with scores between 32 and 47 (*n* = 5), and early chronotypes, with scores between 47 and 55 (*n* = 7). 

### 2.3. Sleep Logs (SL)

Participants were instructed to fill in sleep logs (SL) every morning after getting up during both sample periods. Individual data of the night before the night in which melatonin was measured in Montevideo and Antarctica were extracted from the SL, which was a working day in both locations. Sleep duration was calculated as the difference between sleep end and sleep onset (SO). To measure the impact of Antarctica on bedtime, we calculated the difference between sleep onset values (ΔSO = SOAntarctica – SOMontevideo) for each participant.

### 2.4. Light Recording

Illuminance (in lux, lx) in both site locations (Antarctica and Montevideo) was measured by HOBO® data loggers (8 K, Onset Computer Corporation) placed in clear outdoors areas during all the study period. Participants’ light exposure (in lx) was measured by ambulatory wrist actimeters (GeneActiv Original, Activinsights Ltd., Cambridge, UK) equipped to record it by 1-min epochs. Hourly averages (0–23 h) of light exposure were calculated for each participant during the entire study period in both site locations (Antarctica and Montevideo). 

### 2.5. Melatonin Measurements

Hourly saliva samples (1–2 mL, 18–24 h) were collected in dim light (<30 lx) on the last night of each period (January 26 in Antarctica and March 17 in Montevideo). The timing of the sampling period was fixed for both locations but included sunset in one of the latest saliva samples in Antarctica (sunset at 22 h) and in one of the first samples in Montevideo (sunset at 19 h). Saliva samples were obtained at the same time and in the same conditions for all participants at both the Antarctic base dormitories and our laboratory in Montevideo. Participants remained in resting position except for brief trips to the also dark bathroom and were not allowed to use light-emitting devices during the sampling period. They rinsed their mouth with water before each sample and received a light meal between the 20 h and 21 h samples. Saliva samples were frozen, stored at −20 °C, and later assayed for melatonin using the salivary melatonin competitive ELISA kit from Salimetrics following the manufacturer’s instructions. The tests were carried out by the same technician, at the same time during 3 consecutive days, and the control and treatment samples were combined in each ELISA plate. The concentration-response curves obtained were indistinguishable from those reported by the manufacturer. The same applied to the IC10 values (the melatonin concentrations inhibiting 10% of the absorbance signal observed in the absence of melatonin), taken as an indication of assay sensitivity. This validates the use of the analytical sensitivity value reported by the manufacturer (1.37 pg/mL melatonin). 

Circadian timing was determined by calculating the DLMO as marker of the individual circadian clock and in particular as indicator of the beginning of the internal biological night [16,23,43,44]. Individual basal melatonin level (IBML) was calculated as the average of the early recordings before the nocturnal step in Montevideo (following [45,46]). Individual DLMO onset was calculated as the interpolated point in time at which the quadratic fit surpassed 2 SD above the IBML in the Montevideo samples. For each individual, the melatonin level corresponding to Montevideo DLMO was considered his/her melatonin threshold. Antarctica DLMO was calculated as the time at which the melatonin level reached the individual threshold (modified from [35]). To measure the impact of Antarctica on the daily pattern of melatonin secretion, we calculated the difference between DLMO values (ΔDLMO = DLMOAntarctica – DLMOMontevideo) for each participant.

### 2.6. Statistical Analysis

Light incidence and light exposure data, along with the chronobiological characterization of the study population, are expressed as mean values ± standard deviation, while DLMO values are presented as median ± mean absolute deviation (MAD) throughout the text. Box plots are used in figures to favor the full display of the data. When data did not comply with normality and/or homoscedasticity, statistical comparisons were analyzed by non-parametric tests: The Wilcoxon signed-rank test for comparisons between Montevideo and Antarctica in the same individuals, the Mann–Whitney U test for comparisons across participants, and the Friedman test for comparing paired variables across time. Otherwise, a paired or unpaired Student´s t-test was used. Values of *p* ≤ 0.05 were considered statistically significant.

## 3. Results

The chronobiological characterization of the study population was performed in the peri-equinox in Montevideo with data obtained from two largely validated questionnaires (MEQ and MCTQ [38,39]), whose outputs were significantly correlated (R^2^ = 0.51, *p* = 0.0091). MSFsc (*n* = 12) was, on average, 5.95 ± 0.83 and ranged from 4.67 to 7.58, indicating participants’ lateness. The average value of the MEQ scores (47 ± 8.01, *n* = 12) was used to separate late participants (32 < MEQ scores < 47, *n* = 5) from early participants (47 < MEQ scores < 55, *n* = 7) within the studied sample. The MEQ scores of early and late participants were significantly different (Mann–Whitney U test, *p* = 0.0053).

### 3.1. Light Exposure

**Figure 1:**
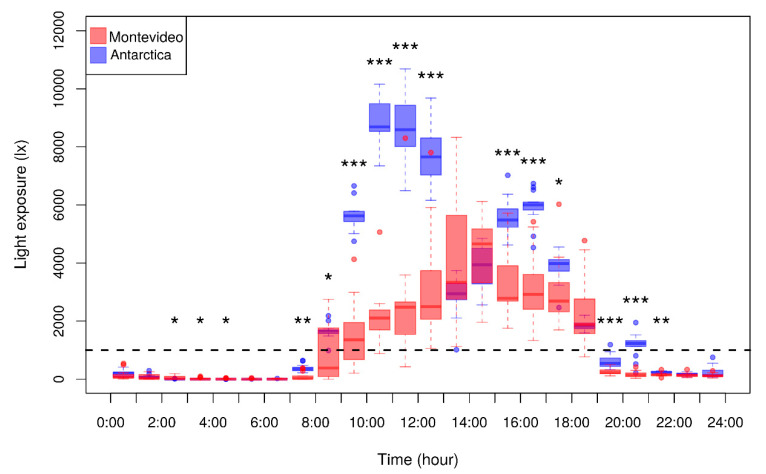
Participants’ hourly light exposure in Montevideo (red) and Antarctica (blue) Light exposure (in lux) was measured by ambulatory wrist actimeters recorded in 1-min epochs. Hourly averages of light exposure were calculated for each participant for nine days in each condition, and hourly data of the whole sample are represented in each box plot. The dotted line represents the 1000 lux threshold that corresponds to outdoor daylight. Asterisks indicate significant hourly differences between Montevideo and Antarctica (paired Student’s t-test; * *p* < 0.05, ** *p* < 0.01, *** *p* < 0.001).

Illuminance during the Antarctic summer was very different with respect to the autumn in Montevideo. Objective data logger measurements confirmed that the daily dark period (<30 lx) lasted 4.58 ± 0.3 h in Antarctica and 11.04 ± 0.14 h in Montevideo, whereas outdoor daylight (>1000 lx) lasted 16.98 ± 0.59 h in Antarctica and 12.15 ± 0.21 h in Montevideo. Consequently, participants were significantly more exposed to light in Antarctica than in Montevideo (Figure 1). Specifically, participants were exposed to a significantly higher light intensity in Antarctica with respect to Montevideo during the early morning (at 7 and 8 h), morning (at 9, 10, 11, and 12 h), afternoon (at 15, 16, and 17 h), and evening (at 19, 20, and 21 h). However, during midday, light exposure was not significantly different between locations (at 13 and 14 h), probably because all participants synchronized their indoor lunchtime in Antarctica. Additionally, very late during the night, light exposure in Montevideo was significantly higher than in Antarctica (at 2, 3, and 4 h), probably because not all the participants slept at the same time in Montevideo, but they did so in Antarctica. During the evening (at 20 h), Antarctic light exposure was not just significantly higher than in Montevideo, it also reached outdoor threshold values (>1000 lx; dotted line in Figure 1). It is also remarkable that participants were exposed to high outdoor light intensity (>5000 lx) only in Antarctica, particularly in the morning. When considering the 3 h-phase-relevant time windows (7:00–9:59, 10:00–12:59, 19:00–21:59, and 22:00–0:59 h), participants were significantly more exposed to light in Antarctica with respect to Montevideo except for the latest time window from 22:00 to 0:59 h (Table 1). Similarly, late participants were significantly more exposed to light in Antarctica with respect to Montevideo in all time windows except the latest one, while early participants were always significantly more exposed to light in Antarctica (Table 1). Interestingly, light exposure was not significantly different between the early and late chronotypes in either time window (Table 1).

### 3.2. Melatonin Levels and DLMO

**Figure 2:**
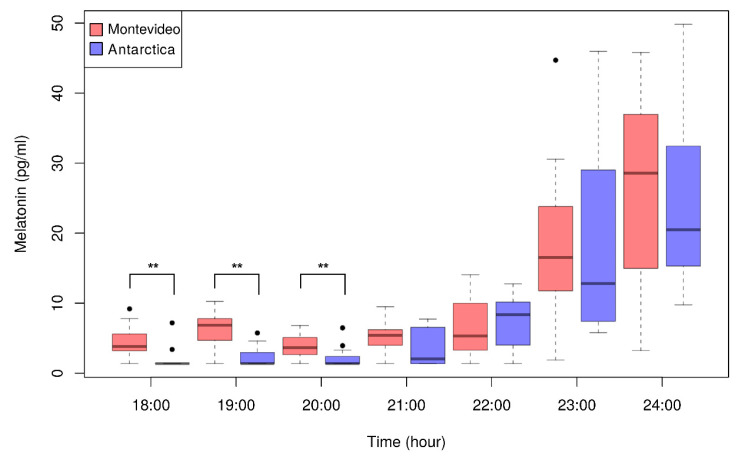
Absolute melatonin levels between from 18:00 to 24:00 in Montevideo (red) and Antarctica (blue). Melatonin levels were obtained at 1-h intervals. The expected nocturnal increase in melatonin levels in both locations was observed. Antarctic diurnal melatonin levels (18:00–21:00 h) were very low (rarely > 7 pg/mL) and significantly different from the respective Montevideo values from 18:00 to 20:00 h (Wilcoxon signed-rank test: ** *p* < 0.01). Black dots represent outliers.

The absolute melatonin levels from 18:00 to 24:00 h in Montevideo and Antarctica are plotted in Figure 2. We observed the expected nocturnal increase in melatonin levels in both locations. Noticeably, Antarctic diurnal melatonin levels (18:00–21:00 h) were very low (rarely >7 pg/mL) and were significantly lower than the respective Montevideo values (asterisks in Figure 2). Consequently, early measurements of melatonin levels (18:00–21:00 h; Figure 2) were more variable in Montevideo than in Antarctica. 

**Figure 3:**
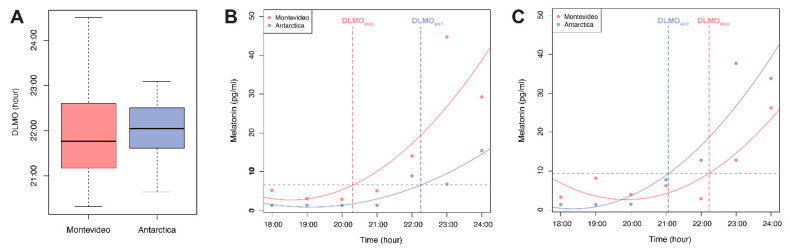
Dim light melatonin onset (DLMO) as a marker of the individual circadian clock. (**A**) DLMO was not significantly different (Wilcoxon signed-rank test, *p* = 0.91) between Montevideo (red) and Antarctica (blue); (**B**,**C**) Examples from two participants showing melatonin levels from 18:00 to 24:00 in Montevideo and Antarctica. The individual DLMO was determined in Montevideo as the interpolated point in time at which the quadratic fit surpassed 2 SD above the basal melatonin level. The abscissa value at this point represented the individual Montevideo DLMO. The ordinate value at this point represented the individual threshold of melatonin levels (horizontal dotted line). The Antarctica DLMO was calculated as the time in which the melatonin levels reached the individual threshold (dotted line). Changes in DLMO between both conditions were different across individuals. (**B**) represents an example of an individual who delayed its DLMO in Antarctica; (**C**) represents an example of an individual who advanced its DLMO in Antarctica.

**Figure 4:**
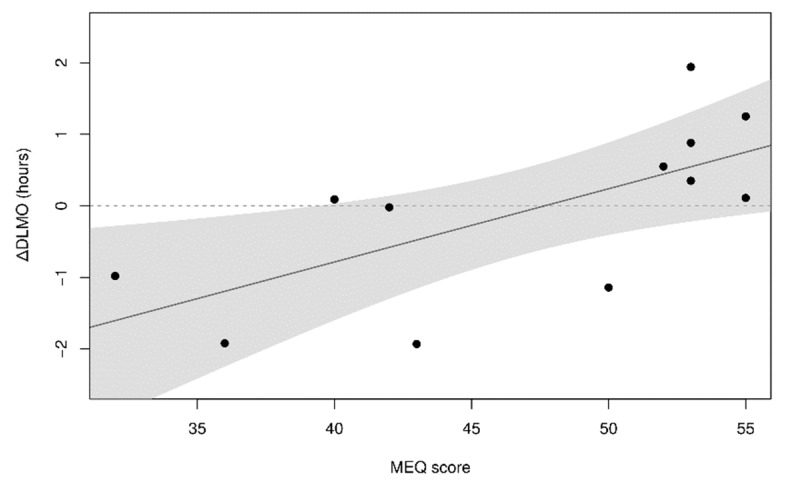
DLMO chronotype-dependence. Individual changes in DLMO between Antarctica and Montevideo (ΔDLMO = DLMOAntarctica – DLMOMontevideo) were correlated with participants’ circadian preferences (R^2^ = 0.441, *p* = 0.019).

As shown in Figure 3A, the DLMO, as marker of the individual circadian clock, was not significantly different between Montevideo (21.77 ± 0.89) and Antarctica (21.20 ± 0.55, Wilcoxon signed-rank test, *p* = 0.91). In agreement with the high variability in the early melatonin, the absolute values observed in Montevideo (Figure 2). DLMO values were also more variable in Montevideo than in Antarctica (Figure 3A). The changes in DLMO between both conditions (ΔDLMO = DLMOAntarctica – DLMOMontevideo) were different across individuals. While five participants delayed their DLMO in Antarctica (shown in an example in Figure 3B and as positive ΔDLMO values in Figure 4), four participants advanced their DLMO in Antarctica (shown in an example in Figure 3C and as negative ΔDLMO values in Figure 4), and three participants did not change their DLMO between sites (ΔDLMO around 0 in Figure 4).

### 3.3. Chronotype-Dependence of Melatonin Levels and Consequences on Sleep Habits

Though Montevideo DLMO values did not correlate with individual circadian preferences (DLMO versus MEQ score, *p* = 0.203), changes in the melatonin profile between Antarctica and Montevideo did. Individual changes in DLMO between Antarctica and Montevideo (ΔDLMO) were correlated with participants’ circadian preferences (R^2^ = 0.441, *p* = 0.019; Figure 4). Earlier chronotypes (high MEQ scores) tended to delay their DLMO in Antarctica with respect to Montevideo (positive ΔDLMO), whereas late chronotypes (low MEQ scores) advanced their DLMO in Antarctica with respect to Montevideo (negative ΔDLMO).

**Figure 5:**
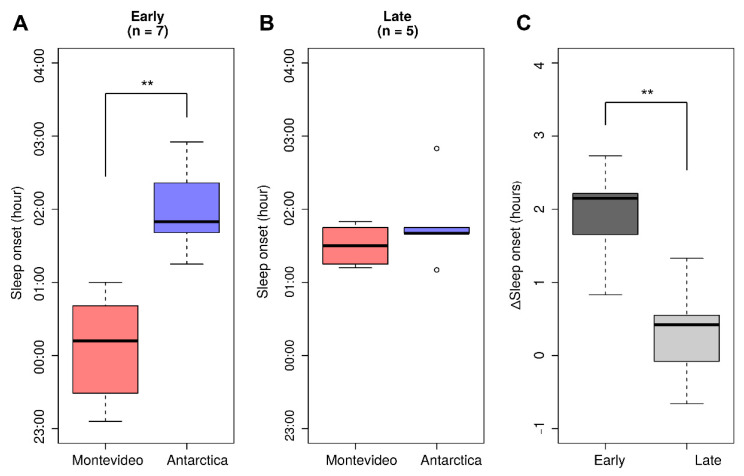
Chronotype-dependent changes in sleep habits between Antarctic and Montevideo. SL data from the last night before melatonin measurements in each condition. (**A**) In early chronotypes, sleep onset (SO) was significantly delayed in Antarctica; (**B**) In late chronotypes, SO did not change between Montevideo and Antarctica. Open circles represent outliers; (**C**) Individual changes in SO between Antarctica and Montevideo (ΔSO = SOAntarctica – SOMontevideo) were significantly different between early and late chronotypes. (** *p* < 0.01).

Participants’ sleep patterns were influenced by Antarctic conditions. When recorded at the end of both sampling periods (the night before saliva sampling), sleep duration was significantly shorter in Antarctica with respect to Montevideo in both early (Wilcoxon signed-rank test, *p* = 0.0179) and late (Wilcoxon signed-rank test, *p* = 0.043) participants. Interestingly, the bedtimes of early and late chronotypes were affected differently by the Antarctic trip (Figure 5). The sleep onset (SO) of early participants was significantly and strongly delayed in Antarctica with respect to Montevideo (Wilcoxon signed-rank test, *p* = 0.0179; Figure 5A), while late chronotypes did not show changes in their SO in Antarctica with respect to Montevideo (Wilcoxon signed-rank test, *p* = 0.5; Figure 5B). In addition, individual changes in SO between Antarctica and Montevideo (ΔSO) were significantly different between early and late chronotypes (Mann–Whitney U test, *p* = 0.0094; Figure 5C).

## 4. Discussion

Though the light modulation of melatonin profile has been largely documented [47,48,49,50], the impact of light on the interactions between sleep patterns, melatonin onset, and circadian preferences is a topic of current debate [35,36]. This study demonstrates the chronotype-dependent effects of changes in light exposure on the melatonin profile and sleep patterns of Uruguayan university students who traveled to Antarctica during the summer. 

The chronobiological characterization of the study population was performed during their normal daily life in the peri-equinox in Montevideo using two validated questionnaires (MCTQ and MEQ [38,39]), whose results were, as expected, significantly correlated [51]. However, while MCTQ provided very late MSFsc values, as previously reported in a similar population [52], circadian preferences derived from MEQ scores were not markedly late. This discrepancy is not surprising because, while the correlation between MEQ and MCTQ is a good indicator of the coherence and quality of the data, both questionnaires have different aims and are not interchangeable [53]. We selected the MEQ score as a proxy of individual circadian preference. The classification of circadian typologies depends on age, geographic, and cultural differences and, thus, cannot be universal [25,54,55]. However, it is acceptable to define cutoffs points to identify earlier and later participants within homogeneous populations as the participants of this study [56]. The use of the average split criterion to categorize participants into two groups [57,58] was validated by the fact that MEQ scores were significantly different between early and late participants. 

Expected but important conclusions emerged from the analysis of both light incidence and participants’ light exposure. A light incidence of high intensity light (>1000 lx) was obviously longer in Antarctica, but, more importantly, participants were actually more exposed to high intensity light in Antarctica than in Montevideo (Figure 1). It is widely recognized that the phase responses of circadian clocks to light could be either advances or delays, depending on the biological time when light stimulation is applied [2,21,59,60,61,62,63,64]. In short, exposure to light during the evening induces phase delays by suppressing nocturnal melatonin secretion [50,63,64,65]. In contrast, exposure to bright light in the morning has been documented to prevent circadian phase shifts and to increase the total amount of nocturnal melatonin secretion [14,15,17,18,20,62]. Therefore, we focused on these sensitive time windows (morning and evening) to evaluate differences in light exposure between Montevideo and Antarctica. Interestingly, we observed that all participants, regardless their chronotype (Table 1), were significantly more exposed to light in Antarctica during the morning (7:00–12:59 h) and at the beginning of the evening (19:00–21:59 h) but not in the late evening time window (22:00–0:59 h). It is interesting to find differences between chronotypes in the light exposure in this late sensitive time window in which 50–100 lx are enough to delay the circadian phase [50]. Both early and late chronotypes were exposed to a similar amount of light from 22:00 to 0:59 h in each location, but a significantly higher light exposure occurred for early participants during the Antarctic trip at this time window (Table 1). 

As the most reliable seasonal time cue is the changing photoperiod, Antarctica—with its extreme high latitude geographical distribution and the confinement and shared schedule of Antarctic bases’ crews—has provided a natural laboratory for the observation and manipulation of seasonal changes in human circadian systems [24]. In the long nights of the Antarctic winter, for example, there is no light information to suppress melatonin levels and thus to set circadian time. Therefore, two lines of evidence arise from Antarctic seasonal studies: 1) Daytime melatonin levels are significantly higher in the Antarctic winter than in the summer [66,67], and 2) the melatonin rhythm is delayed in the winter with respect to the summer [66,68,69,70,71,72]. In addition, a long series of studies conducted in the small overwintering community of the British Halley Research Station demonstrated that the winter delay in the melatonin rhythm can be advanced to the summer phase by bright light treatment [73]. Considering that in the Antarctic summer crew members are more exposed to light in both light-sensitive time windows with opposite effects on circadian shifts, these results indicate that, on average, the morning window is more important in resetting the circadian phase. Experimental data have added evidence to support that bright light in the morning, together with a strong contrast in day–night light, are the most relevant determinants of circadian phase advance [20,61,74,75,76,77,78]. In this regard, as light exposure was enormously higher in Antarctica during all morning time (Figure 1; Table 1), we expected all participants to advance their circadian phase in Antarctica with respect to Montevideo. 

In both experimental conditions (Montevideo and Antarctica), we found the expected melatonin profile with lower levels in daytime and higher levels in nighttime (Figure 2). In line with the well-known suppressor effect of daytime light [13] and the previous Antarctic summer reports [66,73,79], we found that daytime melatonin levels were extremely low in the Antarctic summer and significantly lower than in the peri-equinox in Montevideo (Figure 2). It is currently accepted that DLMO is the best proxy of the individual functioning of the circadian clock, and it is thus a comprehensive way to measure circadian phase shifts [44,80,81,82]. DLMO correlates with circadian preferences [31,32], shows seasonal changes [9,83,84,85], and can be either advanced or delayed by light [20,22,64,78,86]. However, only few previous studies have tested the effects of light changes on the interaction among DLMO, chronotype, and sleep patterns [35,36]. These studies were consistent in showing that DLMO was advanced when participants were more exposed to light in the morning light-sensitive window and that it was delayed when they were more exposed to light in the evening light-sensitive window [35,36]. In contrast to these previous reports, the participants of this study did not show significant changes in DLMO between Montevideo and Antarctica (Figure 3A). This may be due to life conditions in Antarctica that differed not only in light exposure with respect to Montevideo. The confluence of many social confounding interactions is also known to impact on the synchronization of the circadian clock, such as tight schedules, mealtimes or routine physical activity [87,88,89,90,91,92,93]. Indeed, the organized nature of Antarctic life synchronized the circadian system of the participants as daytime melatonin values, DLMO, and awakening times were less variable in Antarctica for the whole study sample.

The lack of an overall DLMO shift between Montevideo and Antarctica did not mean that all individual DLMOs remained unchanged (Figure 3B–C). Rather, participants’ DLMO shifted in a chronotype-dependent manner (Figure 4). Interestingly, although we did not find the expected correlation between DLMO and circadian preferences [32], in Antarctica, late participants tended to advance their DLMO while early participants delayed their DLMO. This chronotype-dependent reversion in DLMO shift may be explained if early participants were significantly more exposed to light than late participants during the evening phase-delay time window or if there was an inter-individual difference in the sensitivity to evening light [94,95]. No differences in Antarctic light exposure between early and late participants were observed at any time (Table 1). However, from 22:00–0:59 h, only early participants were significantly more exposed to light in Antarctica than in Montevideo. Therefore, only early participants were subjected to a differential effect of light during the phase-delaying evening window that was probably enough to induce the observed phase delay. Though it has been previously reported that 200 lx light at the peak of the evening phase-sensitive window delays the circadian phase [50], it is outstanding to note that a subtle increase in light exposure during this late phase-sensitive window (from 100 lx in Montevideo to 200 lx in Antarctica) can counteract the expected effect of the enormous morning light exposure (from 1000 lx in Montevideo to 9000 lx in Antarctica).

Only few previous studies reported chronotype dependent shifts in DLMO. For example, Lewy and Sack [10] found that the later the DLMO (late chronotypes), the higher the phase advance by light in the morning. In addition, Wright et al. [35] reported that the phase advance observed in natural light respect to electric light conditions was higher in late chronotypes. As expected from these observations, we can interpret that late participants of this study were affected by the Antarctic increase in morning light and thus advanced their DLMO, disregarding the effect of the increased light exposure they received during the evening. However, early participants did not respond as expected to the increase in light exposure during the phase-advancing time window, but they did delay their DLMO in Antarctica. Early participants seem to be more responsive to the evening phase-delaying time window than to the morning phase-advancing one. Chronotype differential responsiveness may be explained by the observation that early participants (but not late ones) received a subtle but significant increase in light exposure in Antarctica during the late evening phase-delaying time window. Our data suggest that individual circadian preferences set a differential light history that tunes the susceptibility to light-sensitive time windows. In line with these observations, the Antarctic trip also affected participants’ sleep patterns in a chronotype-dependent manner (Figure 5). As previously reported in a similar population [52], the sleep duration of all participants decreased in Antarctica with respect to Montevideo, probably due to the tight agenda of both social and academic schedules of the Antarctic summer school. However, it is interesting to note that early participants (but not late ones) delayed their sleep onset in Antarctica despite their important sleep debt. We interpret that the light-induced delay in the circadian phase observed in early participants also prevented them from going to sleep earlier to ameliorate their sleep deficit.

## 5. Conclusions

The summer trip to Antarctica meant, for this group of university students (when compared with their normal lives in the peri-equinox in Montevideo), a significant increase in light exposure in both the morning phase-advancing (7:00–12:59 h) and in the evening phase-delaying (19:00–0:59 h) time windows. Though participants with earlier and later circadian preferences were exposed to a similar amount of light in each site and in both time windows, in Antarctica, late chronotypes advanced their DLMO with no changes in sleep onset time and early chronotypes delayed their DLMO and sleep onset time. The behavior of late chronotypes is in line with previous observations in the Antarctic summer [66,68,69,70] and in light manipulation experiments [35,36,73], which demonstrated the powerful circadian phase advancing effects of increasing light in the morning. In contrast, early chronotypes seem to be more responsive to the Antarctic increase in evening light exposure, which delayed their DLMO and thus prevented them from advancing their sleep start times to mitigate their strong sleep deficit. In this natural experiment, we have shown that there is a different susceptibility between chronotypes to respond to light. Despite the fact that all participants were exposed to an enormous light increase in Antarctica during the morning phase-advancing window, early participants delayed their circadian phase and sleep patterns in response to a subtle increase in light exposure during the evening.

## Figures and Tables

**Table 1:** Light exposure during phase-relevant time windows 7:00–9:59, 10:00–12:59, 19:00–21:59, and 22:00–0:59 time intervals for all the participants and separately for early and late chronotypes in both Montevideo and Antarctica locations.

Time Interval (h)	Location	Light Exposure (lx)			
		All Participants	Early Chronotypes	Late Chronotypes	*p*-Value
7:00–9:59	Montevideo	869.13 ± 678.48	1070.66 ± 710.29	587.00 ± 581,89	0.24
	Antarctica	2554.87 ± 187.32	2607.73 ± 177.56	2480.88 ± 193.56	0.27
	*p*-value	0.00002	0.002	0.002	
10:00–12:59	Montevideo	2733.64 ± 1536.76	2943.78 ± 1923.95	2439.46 ± 869.84	0.60
	Antarctica	8459.67 ± 957.59	8787.97 ± 959.46	8000.06 ± 829.56	0.17
	*p*-value	0.00001	0.001	0.0002	
19:00–21:59	Montevideo	189.49 ± 76.38	170.00 ± 68.38	216.77 ± 86.22	0.32
	Antarctica	699.81 ± 207.15	745.41 ± 258.33	653.96 ± 95.76	0.39
	*p*-value	0.00001	0.001	0.004	
22:00–00:59	Montevideo	137.01 ± 91.62	100.41 ± 42.41	188.26 ± 121.49	0.10
	Antarctica	200.12 ± 123.41	231.47 ± 140.14	156.23 ± 91.08	0.32
	*p*-value	0.16	0.044	0.50	

Values are represented as mean ± SD. Light exposure was not significantly different between early and late chronotypes in either time window in both locations (unpaired Student’s t-test).

## Abbreviations

DLMO	Dim Light Melatonin Onset
MEQ	Mornningness-Eveningness Questionnaire
MCTQ	Munich Chrono-Type Questionnaire
MSFsc	Mid-Sleep Free days (sleep corrected)
SO	Sleep Onset
SL	Sleep Log
IBML	Individual Basal Melatonin Level
